# Barriers to Timely Percutaneous Coronary Intervention (PCI) in Rural vs. Urban Settings: A Health System Approach

**DOI:** 10.7759/cureus.97949

**Published:** 2025-11-27

**Authors:** Muhammad Shehryar, Sibtain Nisar, Rashid Murad

**Affiliations:** 1 Department of Cardiology, District Headquarter Teaching Hospital, Mardan, PAK; 2 Department of Cardiology, Peshawar General Hospital, Peshawar, PAK; 3 Department of Gastroenterology, Sheikh Zayed Hospital Lahore, Lahore, PAK

**Keywords:** health services accessibility, myocardial infarction, percutaneous coronary intervention, prehospital care, rural health services, st-elevation, time-to-treatment

## Abstract

Introduction: Timely primary percutaneous coronary intervention (PCI) is crucial for reducing morbidity and mortality in ST-elevation myocardial infarction (STEMI). Delays in rural populations are often driven by geographic, patient-related, and system-level barriers.

Objective: The objective of the study is to evaluate and compare barriers to timely PCI in rural versus urban STEMI patients, identify independent predictors of treatment delay, and quantify their impact on reperfusion times.

Methods: This cross-sectional study enrolled 273 adult STEMI patients undergoing primary PCI at District Headquarter (DHQ) Teaching Hospital, Mardan, over 12 months (137 rural and 136 urban). Patients who received prior fibrinolysis, were referred from other PCI-capable centers, or declined participation were excluded. Patient- and system-level barriers were operationally defined and measured using a structured, pre-tested questionnaire supplemented by medical record review. Patient-level barriers included delayed symptom recognition, transport difficulties, and financial constraints; system-level barriers included off-hour PCI unavailability, inter-facility transfer delays, and referral inefficiencies. Door-to-balloon and symptom-to-door times were recorded and analyzed, adjusting for age, gender, comorbidities, and transport mode. Missing data were handled via listwise deletion. Multivariate logistic regression was conducted after testing model assumptions and assessing multicollinearity to identify independent predictors of PCI delay.

Results: Rural patients had significantly longer door-to-balloon (105.5 ± 15.8 vs. 84.1 ± 11.9 min, p < 0.001) and symptom-to-door (295 ± 80 vs. 145 ± 50 min, p < 0.001) times compared to urban patients. Delays > 90 minutes occurred in 82.5% of rural versus 27.9% of urban patients. Multivariate logistic regression identified rural residence (adjusted odds ratio (AOR) 4.75, 95% confidence interval (CI) 2.95-7.65), patient-related barriers (AOR 2.35, 95% CI 1.50-3.68), and system-related barriers (AOR 2.80, 95% CI 1.85-4.25) as independent predictors of PCI delay.

Conclusion: Rural STEMI patients experience significant delays in receiving primary PCI, primarily due to patient- and system-level barriers. Interventions targeting health education, pre-hospital transport, and referral streamlining are essential to reduce rural-urban disparities and improve clinical outcomes, in line with international guideline recommendations for timely reperfusion.

## Introduction

Cardiovascular diseases (CVDs) remain the leading cause of mortality worldwide, with acute myocardial infarction (AMI) representing one of the most common and life-threatening presentations [1]. Prompt reperfusion therapy, particularly primary percutaneous coronary intervention (PCI), is the gold standard for managing ST-elevation myocardial infarction (STEMI), as it restores coronary perfusion, reduces infarct size, and improves survival [2]. The effectiveness of PCI is highly time-dependent, with both door-to-balloon time and symptom-to-door time being critical metrics. International guidelines, including American College of Cardiology (ACC)/American Heart Association (AHA) recommendations, advise a door-to-balloon time of less than 90 minutes to minimize morbidity and mortality [3]. Delays beyond these windows are associated with increased short- and long-term complications, including heart failure, recurrent ischemic events, and higher mortality [4]. Total ischemic time, combining symptom onset to reperfusion, further emphasizes the importance of timely care.

Despite the proven benefits of PCI, disparities in access persist, particularly between rural and urban healthcare settings [5]. Rural patients often experience longer symptom-to-door and door-to-balloon times due to limited catheterization facilities, under-resourced emergency services, and longer transfer distances to tertiary centers [6]. Urban centers generally benefit from better infrastructure, readily available interventional cardiologists, and PCI-capable facilities, facilitating faster reperfusion [7]. Recent regional data indicate that these disparities contribute to higher in-hospital mortality and poorer long-term outcomes among rural STEMI patients [8].

Barriers to timely PCI are multifactorial, operating at patient, provider, and system levels [9]. Patient-related barriers include delayed symptom recognition, low health literacy, and financial constraints [10]. Provider-level barriers, including delayed diagnosis or off-hour unavailability of trained staff, were considered but not the primary focus of this study [11]. System-level factors encompass fragmented referral networks, inefficient inter-facility transfers, and limited pre-hospital triage capabilities [12]. Health systems in low- and middle-income countries, including Pakistan, face challenges such as under-resourced emergency departments, limited insurance coverage, and infrastructural deficiencies, which disproportionately affect rural populations [8].

Although previous studies have examined delays in PCI, many have focused primarily on patient or clinical factors, without comprehensively addressing broader health system barriers [13]. Comparative analyses of rural versus urban STEMI patients within the same healthcare system are limited, especially in areas with pronounced resource disparities [14]. This study is guided by a health system framework, focusing on institutional- and regional-level factors affecting PCI timeliness. The study hypothesizes that rural residence is associated with increased PCI delays due to patient- and system-level barriers. This study aims to identify and compare the health system barriers leading to delayed primary PCI in rural versus urban settings, providing evidence-based insights to optimize STEMI care pathways and reduce treatment delays.

## Materials and methods

Study design and setting

This cross-sectional comparative study was conducted at the Department of Cardiology, District Headquarter (DHQ) Teaching Hospital, Mardan, a tertiary referral center serving both rural and urban catchment areas. The study spanned 12 months, from July 2023 to June 2024. The study was guided by a health system framework, considering patient-, provider-, and system-level factors affecting timely PCI access. Rural and urban residence was classified according to official administrative definitions: rural patients were those residing in villages or union councils with limited PCI-capable facilities, whereas urban patients were residents of Mardan city or other towns with closer access to tertiary care.

Study population

Inclusion criteria were adult patients (≥18 years) presenting to the emergency department with a confirmed diagnosis of STEMI and scheduled for primary PCI. Exclusion criteria included patients who received fibrinolytic therapy prior to hospital arrival, referrals from other tertiary PCI-capable centers, and patients who refused participation. These criteria ensure that the study population reflects first-contact STEMI patients for whom timely PCI was indicated.

Sample size calculation

Using the WHO formula for a single population proportion [15]:

$\frac{Z^2 \cdot p \cdot (1-p)}{d^2}$

Where n = required sample size, p = 0.204 (estimated prevalence of long pre-hospital delay > 6 hours from a study in Larkana, Pakistan [16]), Z = 1.96 (95% confidence), and d = 0.05 (margin of error). The prevalence from Larkana was considered reasonably comparable due to similar regional healthcare access and socioeconomic profiles. A 10% increase was applied to account for dropouts or incomplete data, yielding 273 participants: 137 rural and 136 urban.

Sampling technique

A consecutive sampling technique was employed. All eligible STEMI patients presenting to the emergency department during the study period were systematically screened, and those meeting the inclusion criteria were enrolled until the target sample size was reached. Eligible participants included adults aged 18 years or older who presented directly to DHQ Teaching Hospital, Mardan, with a confirmed diagnosis of STEMI and were scheduled for primary PCI, had not received fibrinolytic therapy prior to arrival, and provided written informed consent. Patients were excluded if they were referred from other tertiary PCI-capable centers, had incomplete data for essential variables such as symptom-onset or door-to-balloon time, presented with non-STEMI cardiac conditions (e.g., NSTEMI and unstable angina), or declined participation.

Data collection

Data were collected using a structured questionnaire developed based on prior literature and pre-tested on 20 STEMI patients to ensure clarity, relevance, and reliability. The questionnaire was administered by trained research assistants blinded to the patient’s residence status to reduce bias. Clinical and temporal data, including door-to-balloon and symptom-onset times, were verified against hospital records; patient self-report was used only to confirm symptom onset if documentation was incomplete. Barriers were categorized as patient-related (delayed symptom recognition, transport, and financial issues), provider-related (delayed diagnosis, limited staff, and catheterization team delays), and system-related (off-hour PCI unavailability, inter-facility transfer, and referral delays).

Outcome measures

The primary outcome was the occurrence of PCI delay, defined as door-to-balloon time > 90 minutes, and associated barriers. Secondary outcomes were identification of modifiable factors associated with reduced time to reperfusion, including ambulance use, inter-facility transfer, and pre-hospital transport.

Data analysis

Data were entered and analyzed using SPSS v26 (IBM Corp., Armonk, NY, US). Descriptive statistics summarized demographics and clinical variables. Continuous variables were expressed as mean ± SD; categorical variables as n (%). Comparisons between rural and urban groups used t-tests for continuous variables and chi-squared tests for categorical variables. Multivariate logistic regression identified independent predictors of PCI delay, adjusting for confounders: age, gender, comorbidities, transport mode, and residence. Multicollinearity was assessed using the variance inflation factor (VIF), and model fit was evaluated with the Hosmer-Lemeshow test. Missing or incomplete data (<5%) were handled using listwise deletion. No adjustments for multiple comparisons were made, but subgroup analyses were interpreted cautiously.

Ethical considerations

The study was approved by the Institutional Review Board (IRB) of DHQ Teaching Hospital, Mardan. Written informed consent was obtained. Data were securely stored on password-protected computers, with restricted access to the research team. The questionnaire underwent external validation via expert review prior to study initiation. Confidentiality and anonymity were strictly maintained throughout.

## Results

**Figure 1:**
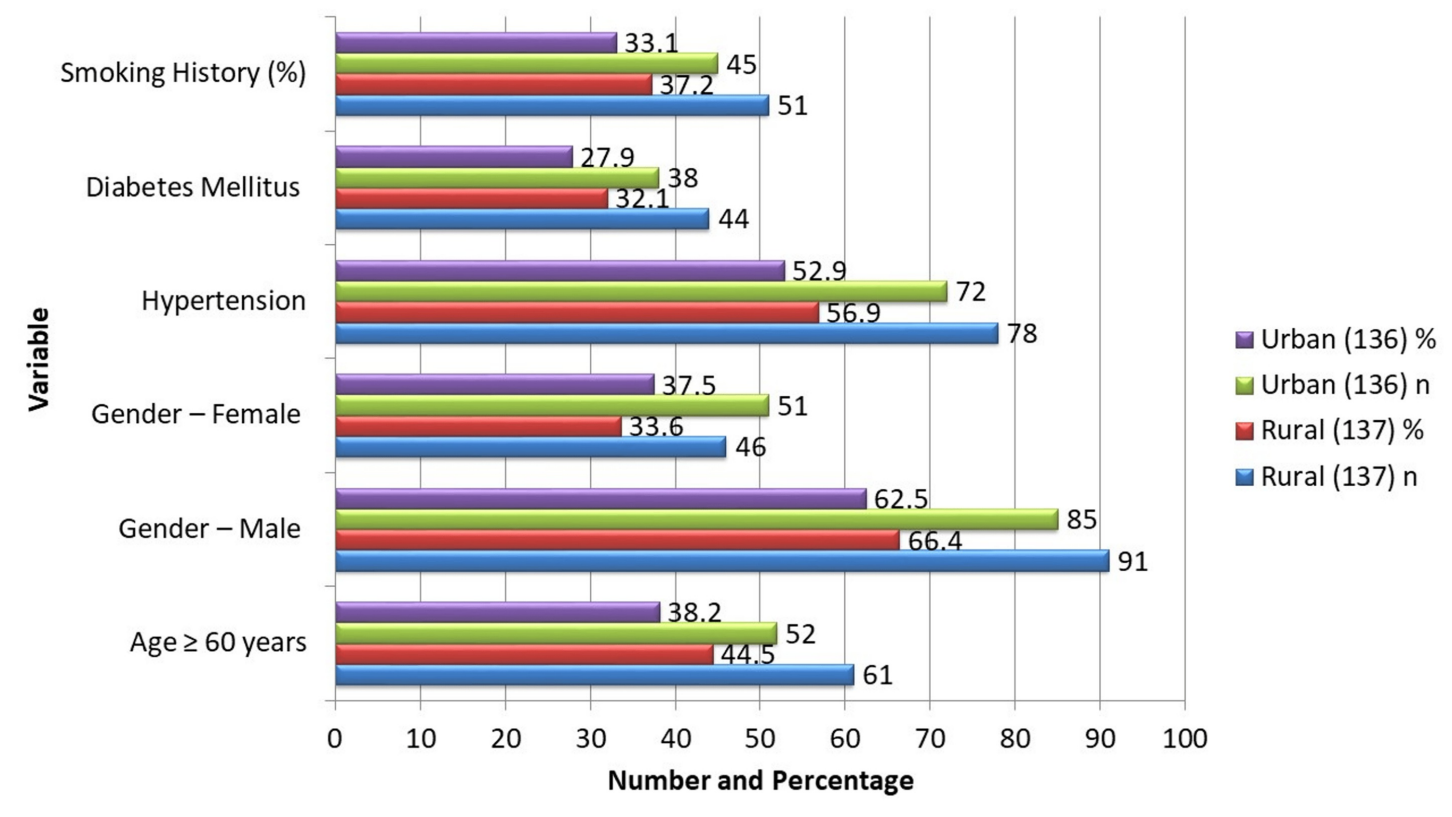
Demographic and Baseline Characteristics of Study Participants. Data are presented as n (%). Comparisons between rural and urban groups used the independent t-test for mean age (t = 1.81, p = 0.07) and chi-squared tests for categorical variables: male gender (χ² = 0.77, p = 0.38), hypertension (χ² = 0.49, p = 0.48), diabetes (χ² = 0.61, p = 0.43), and smoking history (χ² = 0.47, p = 0.49). p < 0.05 was considered significant.

A total of 273 STEMI patients undergoing primary PCI were included: 137 rural and 136 urban. The mean age was 56.1 ± 9.0 years, with no significant difference between rural patients (56.9 ± 9.2) and urban patients (55.3 ± 8.7, t = 1.81, p = 0.07). As illustrated in Figure 1, gender distribution and comorbidities were also comparable: male 91/137 (66.4%) vs. 85/136 (62.5%, χ² = 0.77, p = 0.38), female 46/137 (33.6%) vs. 51/136 (37.5%), hypertension 78/137 (56.9%) vs. 72/136 (52.9%, χ² = 0.49, p = 0.48), diabetes 44/137 (32.1%) vs. 38/136 (27.9%, χ² = 0.61, p = 0.43), and smoking 51/137 (37.2%) vs. 45/136 (33.1%, χ² = 0.47, p = 0.49). These findings indicate that rural and urban participants were similar in baseline demographics and clinical characteristics, minimizing confounding and allowing clearer evaluation of pre-hospital and health system barriers.

As shown in Table 1, rural STEMI patients had significantly longer door-to-balloon times (105.5 ± 15.8 min vs. 84.1 ± 11.9 min, t = 12.02) and symptom-to-door times (295 ± 80 min vs. 145 ± 50 min, t = 17.2) compared to urban patients. PCI delay exceeding 90 minutes occurred in 113/137 (82.5%) rural versus 38/136 (27.9%) urban patients (χ² = 81.24), and PCI delay over 120 minutes in 55/137 (40.1%) rural versus 5/136 (3.7%) urban patients (χ² = 61.7). These results highlight significant rural-urban disparities in timely PCI access. Rural patients had longer symptom-to-door times (295 ± 80 min vs. 145 ± 50 min, t = 17.2) and door-to-balloon times (105.5 ± 15.8 min vs. 84.1 ± 11.9 min, t = 12.02), were less likely to use ambulances (44/137 (32.1%) vs. 89/136 (65.4%), χ² = 30.1), and more frequently required transfers from other facilities (55/137 (40.1%) vs. 21/136 (15.4%), χ² = 22.5). Rural patients had significantly longer symptom-to-door and door-to-balloon times, lower ambulance use, and more inter-facility transfers compared to urban patients (all p < 0.001, Table 1).

**Table 1:** Demographics, Clinical, and Transport Parameters of STEMI Patients. Data are presented as mean ± SD for continuous variables and n (%) for categorical variables. Independent t-tests were applied for continuous variables, and chi-squared tests were used for categorical variables. A p-value of <0.05 was considered statistically significant. All reported p-values in this table indicate statistically significant differences where applicable. STEMI: ST-elevation myocardial infarction; PCI: percutaneous coronary intervention

Parameter	Rural (n = 137)	Urban (n = 136)	Test & value	p-value
Mean age (years ± SD)	56.9 ± 9.2	55.3 ± 8.7	t = 1.81	0.07
Male gender (n, %)	91 (66.4%)	85 (62.5%)	χ² = 0.77	0.38
Female gender (n, %)	46 (33.6%)	51 (37.5%)	χ² = 0.77	0.38
Hypertension (n, %)	78 (56.9%)	72 (52.9%)	χ² = 0.49	0.48
Diabetes (n, %)	44 (32.1%)	38 (27.9%)	χ² = 0.61	0.43
Smoking (n, %)	51 (37.2%)	45 (33.1%)	χ² = 0.47	0.49
Symptom-to-door time (min ± SD)	295 ± 80	145 ± 50	t = 17.2	<0.001
Door-to-balloon time (min ± SD)	105.5 ± 15.8	84.1 ± 11.9	t = 12.02	<0.001
PCI delay > 90 min (n, %)	113 (82.5%)	38 (27.9%)	χ² = 81.24	<0.001
PCI delay > 120 min (n, %)	55 (40.1%)	5 (3.7%)	χ² = 61.7	<0.001
Ambulance use (n, %)	44 (32.1%)	89 (65.4%)	χ² = 30.1	<0.001
Transfer from another facility (n, %)	55 (40.1%)	21 (15.4%)	χ² = 22.5	<0.001

**Figure 2:**
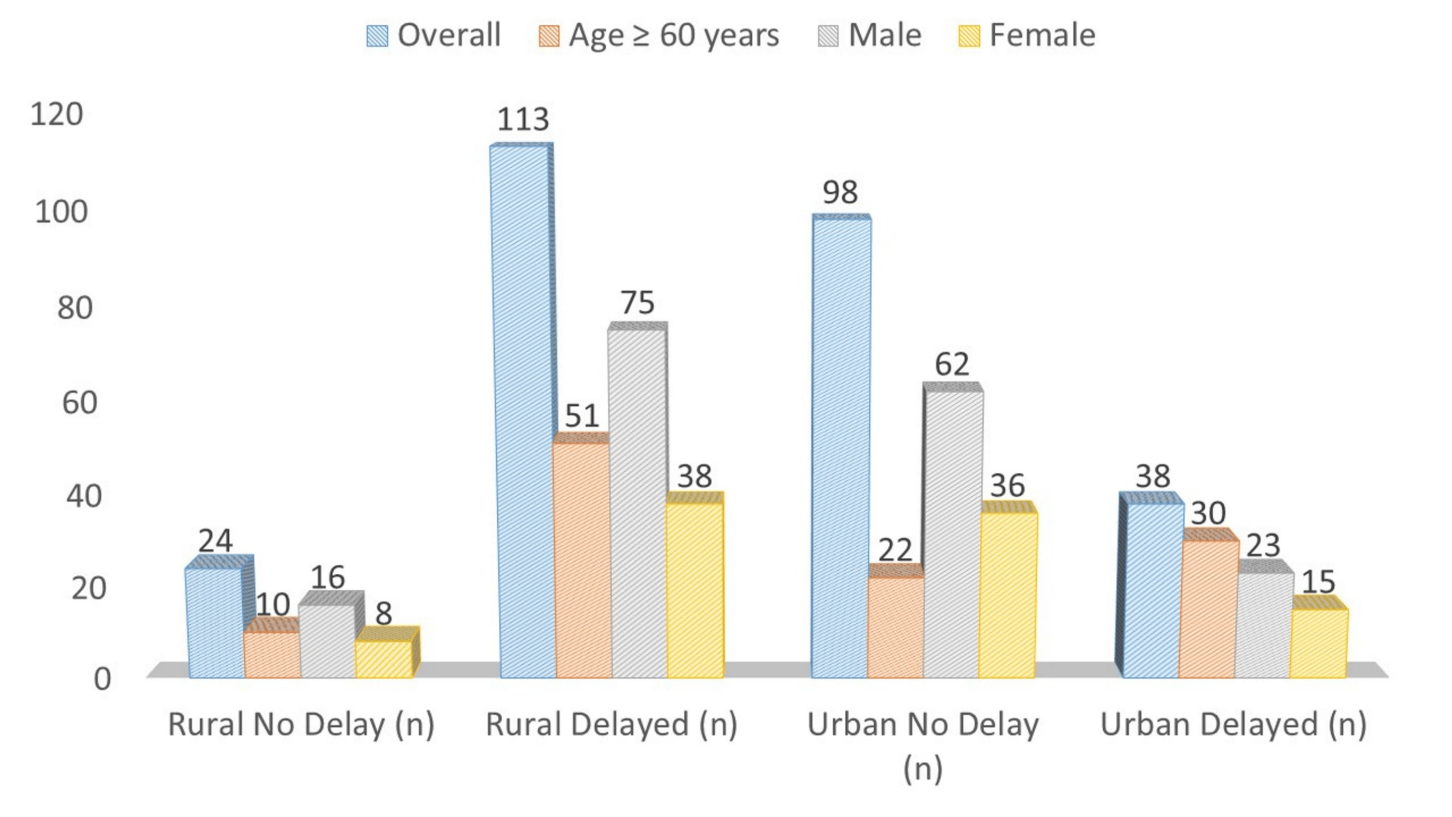
PCI Delay Distribution by Subgroup. Data are presented as frequency (n). PCI delays were higher among rural patients overall (χ² = 81.24, p < 0.001). Significant differences were found for age ≥ 60 years (χ² = 4.12, p = 0.042) and male gender (χ² = 9.76, p = 0.002), while female differences were not significant (χ² = 2.51, p = 0.11). p < 0.05 was considered statistically significant. PCI: percutaneous coronary intervention

PCI delays stratified by age and gender are illustrated in Figure 2. Overall, 113/137 (82.5%) rural patients experienced delays versus 38/136 (27.9%) urban patients (χ² = 81.24, p < 0.001). Among patients aged ≥60 years, delays occurred in 51/61 (83.6%) rural versus 30/52 (57.7%) urban patients (χ² = 4.12, p = 0.042). Male rural patients had more delays (75/91, 82.4%) compared to urban males (23/85, 27.1%; χ² = 9.76, p = 0.002), while the difference in females was not statistically significant (38/46 (82.6%) vs. 15/51 (29.4%); χ² = 2.51, p = 0.11). These findings indicate that age ≥ 60 and male gender are associated with higher PCI delays in rural populations.

Table 2 shows health system barriers among rural and urban patients. Patient-related barriers were reported in 84/137 (61.3%) rural versus 33/136 (24.3%) urban patients (χ² = 41.35, p < 0.001), system-related in 76/137 (55.5%) vs. 34/136 (25.0%; χ² = 30.22, p < 0.001), and provider-related in 58/137 (42.3%) vs. 32/136 (23.5%; χ² = 9.57, p = 0.002). All were significantly higher in rural populations, highlighting the combined impact of patient, provider, and system factors on PCI delays.

**Table 2:** Frequency of Health System Barriers by Specific Categories. Data are presented as n (%). Chi-squared tests applied. p < 0.05 was considered significant. All comparisons were statistically significant.

Barrier type	Specific examples	Rural n (%)	Urban n (%)	Total n (%)	Test & value (χ²)	p-value
Patient-related	Symptom unawareness, transport issues	84 (61.3)	33 (24.3)	117 (42.9)	41.35	<0.001
Provider-related	Delayed diagnosis, staff shortage	58 (42.3)	32 (23.5)	90 (33.0)	9.57	0.002
System-related	Off-hour unavailability, referral delay	76 (55.5)	34 (25.0)	110 (40.3)	30.22	<0.001

Multivariate logistic regression identified rural residence (adjusted odds ratio (AOR) 4.75, 95% confidence interval (CI) 2.95-7.65), patient-related barriers (AOR 2.35, 95% CI 1.50-3.68), and system-related barriers (AOR 2.80, 95% CI 1.85-4.25) as significant predictors of PCI delay. Provider-related barriers showed a borderline effect (AOR 1.50, 95% CI 0.95-2.38). Interaction terms revealed that age ≥ 60 years × rural residence (AOR 1.85, 95% CI 1.10-3.12) and male × rural residence (AOR 1.72, 95% CI 1.02-2.91) further increased the likelihood of delay. Transport-related factors were also significant: ambulance use reduced odds of delay (AOR 0.52, 95% CI 0.31-0.88), while inter-facility transfer increased odds (AOR 2.12, 95% CI 1.25-3.61). Model diagnostics indicated good fit (Hosmer-Lemeshow χ² = 6.21, p = 0.62), good discrimination (receiver operating characteristic area under the curve (ROC AUC) = 0.81), and moderate explanatory power (Nagelkerke R² = 0.42). Multicollinearity was not present (VIF < 2.0), and continuous variables met linearity in the logit assumption. These findings underscore that rural residence, health system barriers, and transport challenges substantially contribute to delayed PCI, which may adversely affect clinical outcomes such as morbidity and mortality. Residual confounding from unmeasured factors (e.g., socioeconomic status) may persist (Table 3).

**Table 3:** Logistic Regression Analysis for Predictors of PCI Delay. Multivariate logistic regression using the Wald test, including interaction terms for age ≥ 60 × rural residence and male × rural residence to assess effect modification on PCI delay. Data are presented as adjusted odds ratios (AOR) with 95% confidence intervals (CI) and p-values. Continuous variables were analyzed using independent t-tests, and categorical variables using chi-squared tests. Hosmer-Lemeshow χ² = 6.21, p = 0.62; ROC AUC = 0.81; Nagelkerke R² = 0.42. Multicollinearity assessed via VIF < 2.0 for all variables. Continuous variables (door-to-balloon and symptom-to-door times) met linearity in the logit assumption. The final model included 273 observations with complete data. p-value < 0.05 was considered statistically significant. Rural residence, patient-related, and system-related barriers were significant predictors of PCI delay. PCI: percutaneous coronary intervention; ROC AUC: receiver operating characteristic area under the curve; VIF: variance inflation factor

Variable	AOR	95% CI	Wald value	p-value
Rural residence	4.75	2.95-7.65	31.8	<0.001
Patient-related barriers	2.35	1.50-3.68	10.2	0.001
Provider-related barriers	1.50	0.95-2.38	3.1	0.08
System-related barriers	2.80	1.85-4.25	16.5	<0.001
Age ≥ 60 × rural residence	1.85	1.10-3.12	5.1	0.024
Male × rural residence	1.72	1.02-2.91	4.0	0.046
Ambulance use	0.52	0.31-0.88	6.5	0.011
Transfer from another facility	2.12	1.25-3.61	8.2	0.004

## Discussion

This study demonstrated significant disparities in timely PCI between rural and urban STEMI patients in Mardan. Rural patients experienced markedly longer door-to-balloon and symptom-to-door times and a higher prevalence of delays exceeding 90 and 120 minutes and were more likely to encounter patient-related, provider-related, and system-related barriers. Multivariate logistic regression, adjusting for transport and demographic variables, identified rural residence, patient-related, and system-related barriers as independent predictors of PCI delay. Provider-related barriers showed a borderline effect, suggesting that while staffing delays and off-hour availability may contribute to treatment delays, their impact is less pronounced once patient and system factors are considered.

These findings expand upon prior regional studies in Pakistan and international literature, demonstrating that geographic, demographic, and health system determinants consistently influence PCI delays [3-5]. Unlike some urban-focused studies reporting minimal delays once PCI services are available [2], our results highlight persistent rural-urban inequities even after adjusting for ambulance use, inter-facility transfers, age, and comorbidities. The effect of rural residence remained significant after controlling for these variables, emphasizing structural limitations in rural healthcare delivery.

Patient-related barriers, including delayed symptom recognition and late presentation, align with previous evidence emphasizing the role of health literacy and awareness in pre-hospital delay [17]. System-level factors-including off-hour PCI unavailability, limited pre-hospital ECG capability, absence of telecardiology, and referral delays-further exacerbate delays, highlighting the impact of health infrastructure limitations on timely STEMI care in Mardan [18]. Seasonal or temporal variations over the 12-month study period did not significantly influence delay patterns, suggesting that structural rather than temporal factors predominantly drive disparities. Urban patients, in contrast, benefited from shorter travel distances, better ambulance access, and streamlined referral pathways, explaining significantly shorter door-to-balloon times [7,19].

Older patients and males in rural areas experienced higher delays, likely reflecting comorbidity-related decision complexity and social factors such as occupational responsibilities [20]. These findings emphasize that both individual and systemic determinants must be addressed to improve timely PCI access. The potential for reverse causality and recall bias in reporting symptom onset times is acknowledged, as patients’ recollection may be imperfect. Additionally, unmeasured confounders, including socioeconomic status, education, and healthcare-seeking behavior, may have influenced outcomes.

Interventions to reduce rural-urban disparities should be context-specific. Feasible strategies in Mardan may include public awareness campaigns targeting symptom recognition, pre-hospital triage with ambulance-based ECG, and integration of telecardiology systems to support early diagnosis and expedite transfers. Resource allocation to expand off-hour PCI services and strengthen inter-facility referral pathways could further improve timely reperfusion. These measures align with health policy objectives to enhance equity in cardiovascular care, particularly in under-resourced rural areas [6,10].

Comparisons with national PCI benchmark data indicate that while urban centers in Pakistan generally achieve door-to-balloon times within guideline-recommended thresholds, rural populations frequently exceed these targets [5,21]. Our study contributes evidence-based insights to guide policy decisions, emphasizing the need for systemic improvements and targeted interventions to reduce delays in STEMI management.

Limitations and future suggestions

This study provides important insights into rural-urban disparities in PCI access; however, several limitations should be acknowledged. Being conducted at a single tertiary hospital may limit the generalizability of findings to other regions or healthcare settings. Data on certain socioeconomic and behavioral factors, including income, education, and healthcare-seeking behavior, were not collected, which may have influenced delays. Additionally, the reliance on patient-reported symptom onset introduces potential recall bias. Future research should consider multicenter studies with larger and more diverse samples, incorporating both quantitative and qualitative methods to comprehensively assess patient- and system-level barriers. Context-specific interventions-such as targeted health education programs, enhanced pre-hospital transport systems, telecardiology integration, and streamlined referral protocols-should be evaluated for their effectiveness in reducing treatment delays and improving outcomes in rural populations.

## Conclusions

This study demonstrates that rural residence, patient-related factors, and system-level barriers are significant independent predictors of delayed primary PCI in STEMI patients, based on adjusted multivariate regression analysis. Targeted interventions-including community-level health education to improve symptom recognition, institutional improvements in pre-hospital transport and ambulance triage, and policy-level support for streamlined referral and telecardiology systems-have the potential to reduce treatment delays, morbidity, and mortality in rural populations. While findings are derived from a single-center study and may be influenced by unmeasured confounders, they provide actionable insights to guide equitable cardiac care. Future multicenter studies are warranted to validate and expand these results across broader populations.
